# A shield of phosphorylation: MPK3/6 protect STOP1 from PUB24-mediated degradation under hypoxia

**DOI:** 10.1093/plcell/koaf270

**Published:** 2025-11-07

**Authors:** Jiajun Wang, Yueyao Wang

**Affiliations:** Assistant Features Editor, The Plant Cell, American Society of Plant Biologists; School of Life Sciences, Xiamen Key Laboratory of Plant Genetics, Xiamen University, Xiamen 361102, China; School of Advanced Agricultural Sciences, Peking University, Beijing 100871, China

With the intensification of global climate change, flooding-induced hypoxia stress has become increasingly frequent, posing a serious threat to food security. Oxygen deprivation, or hypoxia, is a common stress encountered by plants under conditions such as flooding, waterlogging, or poorly aerated soils and strongly affects plant growth, development, and metabolism. Hypoxia inhibits aerobic respiration, resulting in energy imbalance and excessive accumulation of reactive oxygen species (ROS), which compromises plant survival through both energy deficiency and oxidative toxicity (Wang et al. 2025b). The C2H2-type zinc-finger transcription factor SENSITIVE TO PROTON RHIZOTOXICITY 1 (STOP1) plays a crucial role in plant tolerance to multiple stresses, including aluminum toxicity, phosphate deficiency, and hypoxia (Sadhukhan et al. 2021). In a new study, Jian-Hong Wang, Ying Zhou, and colleagues (Wang et al. 2025a) elucidated how STOP1 responds to hypoxic signals and regulates hypoxia responses in *Arabidopsis thaliana*.

The authors found that hypoxia treatment led to an accumulation of STOP1 protein, whereas its levels rapidly declined during reoxygenation. Overexpression of STOP1 enhanced plant tolerance to hypoxia, while the *stop1-2* mutant was hypersensitive. To uncover the mechanism underlying hypoxia-induced STOP1 stabilization, the authors analyzed public coexpression databases and identified *PLANT U-BOX-TYPE E3 UBIQUITIN LIGASE 23* (*PUB23*) as coexpressed with *STOP1*. Using yeast 2-hybrid, bimolecular fluorescence complementation, pull-down, and co-immunoprecipitation assays, they demonstrated that STOP1 specifically interacts with PUB24. Notably, PUB24 protein abundance decreased under hypoxia but increased upon reoxygenation, exhibiting an opposite pattern to that of STOP1. Further in vivo and in vitro ubiquitination assays confirmed that PUB24 promotes the ubiquitination and degradation of STOP1. Overexpression of PUB24 accelerated STOP1 degradation, whereas the *pub24-1* mutant markedly delayed it. Consistently, *pub24* mutants showed enhanced hypoxia tolerance, while *PUB24* overexpression reduced tolerance. The *stop1 pub24* double mutant was as sensitive as *stop1*, indicating that STOP1 functions downstream of PUB24.

Given previous findings that MPK3 and MPK6 phosphorylate and stabilize STOP1 to regulate auxin-mediated maintenance of stem cell niche identity (Liu et al. 2024) and that MPK3 and MPK6 enhance plant tolerance to hypoxia with their phosphorylation being hypoxia inducible (Zhou et al. 2022), the authors investigated whether MPK3/6 mediate STOP1 phosphorylation and stabilization under hypoxia. Pull-down, bimolecular fluorescence complementation, and co-immunoprecipitation assays confirmed direct interactions between STOP1 and MPK3/6. In vitro kinase assays further revealed that MPK3 and MPK6 phosphorylate STOP1 mainly at 3 residues: Thr386, Ser448, and Ser486. Importantly, hypoxia markedly enhanced the interaction between MPK3/6 and STOP1 in wild-type Arabidopsis protoplasts, and both hypoxia-induced STOP1 phosphorylation and protein accumulation were dependent on MPK3 and MPK6.

To test whether STOP1 phosphorylation affects PUB24-mediated ubiquitination, the authors generated phosphodead and phosphomimic variants of STOP1. The phosphodead STOP1 displayed stronger interaction with PUB24, higher ubiquitination levels, faster protein degradation, and increased hypoxia sensitivity in transgenic plants. In contrast, the phosphomimic variant showed slightly weaker PUB24 interaction, reduced ubiquitination, enhanced protein stability, and improved hypoxia tolerance.

Since phosphatidic acid (PA) levels are known to increase under hypoxia and to promote MPK3/6-mediated phosphorylation (Zhou et al. 2022), the authors investigated whether PA similarly modulates the MPK3/6–STOP1 module. In vitro lipid-binding assays demonstrated that STOP1 binds PA via 3 KR-R residues. Pull-down assays further revealed that PA markedly enhanced the interaction between MPK3/6 and STOP1. In the *pldα1 pldδ* double mutant, which is defective in PA production, the MPK3/6–STOP1 interaction was strongly reduced. Consistently, exogenous PA treatment promoted the accumulation of wild-type and phosphomimic STOP1 proteins, but not of the phosphodead variant.

Beyond stabilizing STOP1, phosphorylation also affected its subcellular localization and transcriptional activity. Further assays showed hypoxia promoted nuclear accumulation of STOP1 and that phosphorylated STOP1 enhanced transcription of key metabolic genes *GDH1* and *GDH2*. Together, the findings reveal a finely tuned regulatory mechanism: under normoxia (normal oxygen levels), PUB24 mediates STOP1 ubiquitination and degradation, whereas under hypoxia, PA promotes the interaction between STOP1 and MPK3/6, enhancing STOP1 phosphorylation and suppressing PUB24-mediated ubiquitination. Meanwhile, hypoxia also decreases PUB24 protein abundance, collectively leading to nuclear accumulation of STOP1 and activation of *GDH1* and *GDH2* transcription. This signaling module enables plants to adjust metabolism and sustain cellular homeostasis under oxygen deprivation (Fig.).

**Figure.**
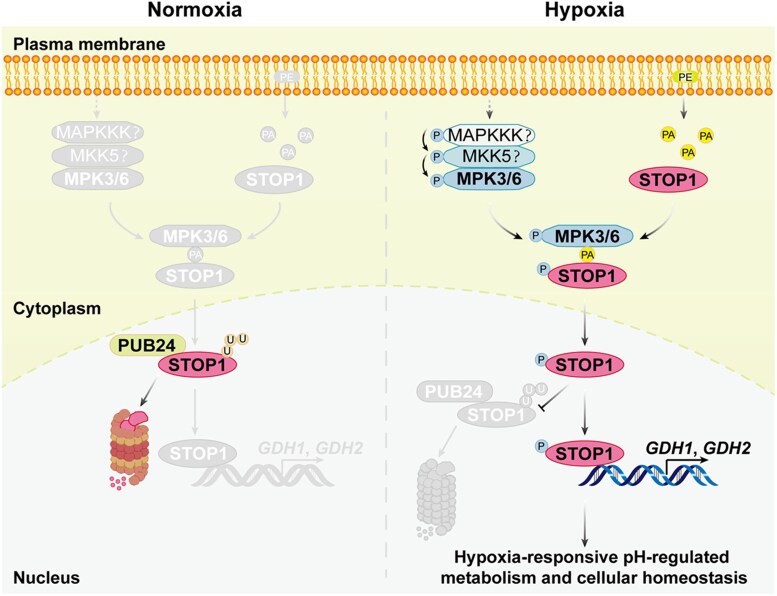
A working model illustrating how MPK3/6-mediated phosphorylation of STOP1 inhibits its ubiquitination by PUB24, thereby promoting nuclear accumulation and regulating hypoxia responses. Under hypoxic conditions, activated MPK3 and MPK6 interact with STOP1, and this interaction is further strengthened by hypoxia-induced PA. Phosphorylation of STOP1 by MPK3/6 suppresses PUB24-mediated ubiquitination and degradation, resulting in STOP1 accumulation in the nucleus. Nuclear STOP1 activates the expression of *GDH1* and *GDH2*, which modulate pH-dependent metabolic processes and help maintain cellular homeostasis under hypoxia. Under normoxia or upon reoxygenation, STOP1 remains predominantly unphosphorylated, becomes ubiquitinated by PUB24 in the nucleus, and is subsequently degraded by the 26S proteasome, thereby attenuating hypoxia responses. Reprinted from Wang et al. (2025a), Figure 9.

## Recent related articles in *The Plant Cell*:

- Wei et al. (2024) revealed that hydrogen peroxide (H₂O₂) negatively regulates aluminum resistance by oxidizing the transcription factor STOP1, which promotes its degradation via the F-box protein RAE1, thereby defining a crucial signaling pathway linking reactive oxygen species to aluminum stress response.
- Yu et al. (2024) identified the calcium-dependent protein kinase CPK16 as a key negative regulator of hypoxia tolerance, which shapes ROS bursts by phosphorylating RBOHD under hypoxia and reoxygenation stress.
- Mou et al. (2024) uncovered that the OsCPK17-OsPUB12-OsRLCK176 module maintains immune homeostasis in rice, where OsCPK17 phosphorylates and stabilizes OsRLCK176 by blocking its OsPUB12-mediated ubiquitination, thereby fine-tuning immunity.
- Zhang et al. (2024) demonstrated that the mitogen-activated protein kinases GmMPK3 and GmMPK6 phosphorylate and stabilize the receptor-like cytoplasmic kinase GmCDL1, forming a positive feedback loop that is activated by nematode infection to regulate soybean basal immunity.

## Dive Curated Terms

The following phenotypic, genotypic, and functional terms are of significance to the work described in this paper:

STOP1 Gramene: AT1G34370
STOP1 Araport: AT1G34370

PUB24 Gramene: AT3G11840
PUB24 Araport: AT3G11840

CPK16 Gramene: AT2G17890
CPK16 Araport: AT2G17890

GDH1 Gramene: AT5G18170
GDH1 Araport: AT5G18170

GDH2 Gramene: AT5G07440
GDH2 Araport: AT5G07440

PUB23 Gramene: AT2G35930
PUB23 Araport: AT2G35930

MPK3 Gramene: AT3G45640
MPK3 Araport: AT3G45640

MPK6 Gramene: AT2G43790
MPK6 Araport: AT2G43790
